# Stress Levels and Mental Well-Being among Slovak Students during e-Learning in the COVID-19 Pandemic

**DOI:** 10.3390/healthcare9101356

**Published:** 2021-10-12

**Authors:** Anna Rutkowska, David Liska, Błażej Cieślik, Adam Wrzeciono, Jaroslav Broďáni, Miroslava Barcalová, Daniel Gurín, Sebastian Rutkowski

**Affiliations:** 1 Faculty of Physical Education and Physiotherapy, Opole University of Technology, 45-758 Opole, Poland; a.rutkowska@po.edu.pl; 2 Department of Physical Education and Sports, Matej Bel University, 97401 Banská Bystrica, Slovakia; david.liska@umb.sk; 3 Faculty of Health Sciences, Jan Dlugosz University in Czestochowa, 42-200 Czestochowa, Poland; b.cieslik@ujd.edu.pl; 4 Department of Physiotherapy, University School of Physical Education in Wrocław, 51-612 Wrocław, Poland; adam.wrzeciono@student.po.edu.pl; 5 Department of Physical Education and Sports, Faculty of Education, Constantine the Philosopher University in Nitra, 94901 Nitra-Chrenová, Slovakia; jbrodani@ukf.sk; 6 Department of Physical Education, Technical University of Košice, 04200 Košice, Slovakia; miroslava.barcalova@tuke.sk; 7 Faculty of Healthcare, Slovak Medical University in Bratislava, 87405 Banská Bystrica, Slovakia; daniel.gurin@szu.sk

**Keywords:** COVID-19, mental health, depression, anxiety, e-learning, Slovakia

## Abstract

The SARS-CoV-2 virus pandemic has forced far-reaching changes in higher education. Isolation from peers and distance learning have significantly limited interpersonal contacts, which might have affected the mental well-being of students. Therefore, the aim of the study was to investigate the prevalence of depressive symptoms and the level of perceived stress during e-learning among Slovak students and to identify the variables that have the most significant impact on mental health among students. The study included 3051 participants, 1773 women (58%) and 1278 (42%) with a mean age of 22.37 years. The Perceived Stress Scale (PSS-10) and Beck Depression Inventory (BDI-II) were used to measure the severity of stress and depression level. In addition, an author’s survey was used assessing the areas of social life, education skills, economic field, nutrition habits, and drugs. Almost all study participants were characterized by increased stress level and 47% of them were depressed. Moreover, isolation affected women more, especially in terms of social life and economics. It seems necessary to implement appropriate support programs for students, which could have the potential to improve their psychological condition.

## 1. Introduction

Coronavirus 2019 SARS-CoV-2 that causes COVID-19 disease has spread rapidly around the world, leading to a global crisis in health systems management, economics, as well as in education [1]. Due to the rapid spread of COVID-19 between humans, most governments have instituted restrictions to prevent the uncontrolled spread of the virus. The outbreak of the COVID-19 pandemic and the prolonged period of restrictions have exposed the problems that society has long experienced. 

Slovakia is one of the countries that has been severely affected by the COVID-19 pandemic. Several restriction mechanisms were implemented at the time of the study: a curfew was imposed on citizens, gatherings of more than six people were forbidden, likewise, all mass events were prohibited. Sports competitions were limited and allowed without an audience, as well as all fitness and wellness centers had to be closed. Kindergartens, primary and secondary schools have been closed, while e-learning has been introduced at all universities, and state exams were conducted online [2].

Introducing restrictions on human contact has been effective in preventing the uncontrolled spread of COVID-19 [3]; however, it appears to have negatively impacted mental health [4,5,6]. Mental health problems have become a major public health concern worldwide [7]. Socially isolated young people, condemned to constant contact with the internet, find themselves in sorrowful situations where the satisfaction of their basic needs (the need for natural, unrestrained social contacts) was impossible. This situation evokes fear and insecurity, which leads to a range of heightened reactions such as stress, post-traumatic stress disorder (PTSD), anxiety, depression, behavioral changes, sleep problems, eating disorders, alcohol and drug abuse [8,9,10,11,12,13]. It would seem that feelings of stress and fear are similarly distributed throughout the population. Research shows that women in threatening situations have higher social sensitivities, reporting more depressive and anxiety symptoms and higher levels of stress than men. Men showed more problems such as substance abuse and internet addiction. The reason for this may be physiological differences between men and women (such as genetic predisposition, hormone levels) and may be reflected emotionally and behaviorally [14,15]. The student population, even before the pandemic, was reported to be at risk for mental health problems relative to other groups [16,17,18,19]. College students represent a population that is particularly vulnerable to mental health disorders in the context of the challenges associated with their progression into adulthood, independence, and grappling with frequent economic and material difficulties [20]. Mental disorders can affect students’ motivation and social interactions - key factors for success in higher education [21,22]. Remarkably, many mental problems may continue long after the pandemic is over, and some may begin to emerge in the near future. Therefore, it is important to recognize these alarming signs among young people.

There have been significant changes in the education system in 2020 where, in fear of a spreading pandemic, most universities in the world have started remote learning. Students were not prepared for the unexpected changes and the e-learning process appeared to be highly stressful. Platforms such as Zoom, Moodle, Blackboard, and Skype have been adapted to deliver content to students. The digitization introduced has caused confusion for both students and academic staff. At this point, however, it is difficult to assess whether the benefits of online classes in the form of easy access (even during the quarantine period) can balance the difficulties, especially those involving practical or even clinical aspects [23]. Previous studies have reported that university students report high levels of stress, anxiety, and depression [24,25], which affects student motivation and attitudes toward learning [26,27].

Young people are at higher risk of developing mental health disorders. Approximately 75% of all psychiatric disorders arise before the age of 24 [28]. Early detection and implementation of therapeutic interventions are crucial. A study by Hajduk et al. in 2019 demonstrated that 35.5% of Slovak students presented symptoms of depression [22]. The gaps in mental health and social care also have been noted in Slovaks among 15–64 year-olds. Two-thirds of people with depressive disorders and more than 80% of people with anxiety disorders did not receive sufficient support and treatment [29]. Thus, it is important to monitor the mental health of young people in an era of the ongoing COVID-19 pandemic; however, to date no such studies have been conducted on a population of Slovakian students.

Therefore, this study was aimed at investigating the prevalence of depressive symptoms and the level of perceived stress during e-learning among Slovak students and to identify the variables that have the most significant impact on mental health among students. The hypotheses were as follows: (H1) Slovak students present symptoms of depression and anxiety and stress; (H2) mental health disorders in the study group affect women more than men.; (H3) the COVID-19 pandemic and implementation of e-learning into the education system has had a negative impact on the areas of social life, educational skills, economics, and nutritional and drug habits among Slovak students.

## 2. Materials and Methods

### 2.1. Participants

The 3051 students were enrolled with sociodemographic characteristics as presented in Table 1. Students represented a total of four Slovak universities: Matej Bel University, Constantine the Philosopher University in Nitra, the Technical University of Košice, and the Slovak Medical University in Banská Bystrica. Information about the study was provided through social media and e-learning platforms. A total of 23,822 students were provided with the survey link, and 12.8% of students decided to participate in the study. All participants agreed to participate in the study and informed consent was obtained. 

### 2.2. Outcome Measures

The questionnaire was compiled using the online Google Forms during the e-learning semester in March and April of 2021.

#### 2.2.1. Perceived Stress Scale (PSS-10)

The PSS-10 is widely used for measuring psychological distress. It contains ten questions on a five-point scale from 0 to 4 [30]. Participants are asked to rate their stress levels over the past month. The higher the score, the greater the feeling of stress. The scale demonstrated a satisfactory internal consistency (Cronbach’s alpha = 0.69).

#### 2.2.2. Beck Depression Inventory (BDI)

The BDI-II is a tool used to determine the degree of intensity of depressive symptoms. The 21-item questionnaire consists of two parts: emotional and somatic. Depending on the number of points obtained, the severity of depression can be assessed. A range of 0–10 points indicates no depression, 11–27 indicates moderate mood disorder, and 28 indicates major depressive disorder. The cutoff for dividing patients into depressive and non-depressive subgroups was a score of 10, following the guidelines given by Beck et al. [31,32]. Internal consistency was high as indicated by Cronbach’s alpha of 0.91.

#### 2.2.3. Authors Survey

The survey contains four sections on the impact of e-learning on various aspects of life. The survey responses were structured on a 5-point Likert scale from 1 to 5, where 1 refers to “strongly disagree” and 5 to “definitely agree”. Questions were grouped into areas: social life (3 questions), education skills (4 questions), economic field (2 questions), nutrition habits and drugs (2 questions). The questionnaire showed satisfactory reliability with a Cronbach alpha of 0.82.

### 2.3. Statistical Analysis

Data were analyzed using SPSS (Version 24.0, SPSS Inc., Chicago, IL, USA). Demographic characteristics, depression, and stress levels were presented with means, standard deviations (SD), and percentages. Percentages of responses to other questions were calculated according to the number of respondents per response to the number of total responses of a question and presented as categorical variables. The unpaired *t* test for independence was used to compare results between genders. The differences in categorical variables were tested using the chi-square test. The required sample size for this study was calculated using the following formula:

$$n=Z2p\times qN\;/\;e^{2}\;\left(N\;-1\right)+Z2p\times q\;$$

(*n* = sample size, *N* = population size, Z = confidence level, *p* = probability of success, *q* = probability of failure, *e* = confidence interval). The confidence level was set at 99%, the confidence interval at 5%, the probability of success at 50%, and the estimated population of Slovak students at 134,000. Therefore, a sample of 384 subjects was required. However, to obtain the most accurate results, it was decided not to block access to the survey once the required sample size was reached. Four variables were tested as possible mediators of the relations between gender and mental health outcomes. Mediators were tested by calculating bias-corrected 95% CI using bootstrapping using the PROCESS function v4.0 in SPSS. Model 4 (model as a parameter in the PROCESS function) was used for the parallel mediation model [33]. Variables were constructed as independent single mediator models. These models were based on studied areas (social life, education skill, economic field, and nutrition habits and drugs). For each mediator, the result of all questions in the model was summed up. The results were presented as the effect size of the total, direct, and indirect effects. The α level was set at 0.05 for statistical significance.

## 3. Results

The study included 3051 participants consisting of 1773 women (58%) and 1278 men (42%) with a mean age of 22.37 (4.22) years. Most of the respondents attended bachelor programs (70%) and 95% studied remotely. Almost 15% preferred e-learning as a study form. Table 2 illustrates the characteristics of the participants. 

For the entire study group, the mean stress level score was 20.85 (5.63) and the depression level was 14.35 (10.22). There were statistically significant differences between the genders, both in the level of stress and depression symptoms. The mean PSS-10 result was 21.97 for women (5.39), and 19.30 for men (5.59); the difference was approximately 12% (*p* < 0.001). In the case of BDI, the difference was approximately 17% (15.43 vs. 12.85, *p* < 0.001) (Table 3.).

**Figure 1:**
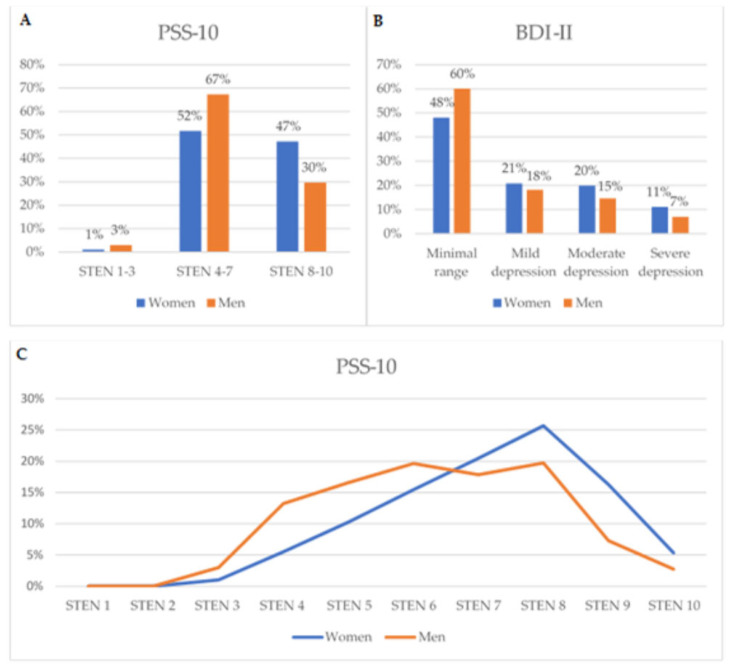
Percentage distribution of participants in the specified cutoffs for PSS-10 (**A**,**C**) and BDI-II (**B**).

Percentage distribution of participants in the specified cutoffs and sten scores for PSS-10 and BDI was presented in Figure 1. Analyzing the PSS-10 sten results, it was shown that most of the subjects were in the 4–10 stens (98%). More men were in the 4–7 stens (52% vs 67%), and the women in the 8–10 stens (47% vs 30%, chi^2^ = 103.61, *p* < 0.001). Over 50% of women and 40% of men indicated symptoms of depression, and this difference was statistically significant (chi^2^ = 46.45, *p* < 0.001). Twenty-one percent of women had mild depression, 20% had moderate, and 11% had severe depression. At the same time, men scored 18%, 15%, and 7% severity for mild, moderate, and severe depression, respectively (Figure 1).

Analyzing the components of the author’s survey, it was found that 83% of participants felt limited social contact, 69% felt a sense of isolation, 68% felt a worsening of practical skills, and 62% believed they experienced a deteriorated quality of education (Table 4). On the other hand, 79% of the respondents stated that their diet did not deteriorate during e-learning. Analyzing the differences between the sexes, women felt a significantly greater sense of isolation (*p* = 0.004), worsening practical skills (*p* = 0.002), a deteriorated quality of education (*p* < 0.001), a limitation of work (*p* < 0.001,) reduced financial resources (*p* < 0.001), increased conflicts within the family (*p* < 0.001), and limited social contact (*p* = 0.007) (Table 4). 

For the mediation analyses, out of the four tested models, two of them, i.e., social and economic models, were included in the final analysis (Table 5). The education model and the drugs and diet model were excluded from the analyses as these were not found to correlate with the other variables. The analysis showed that men experienced a lower severity of depressive symptoms (−2.67) and lower stress levels (−2.58). From the models included in the analysis, the social life model explained 18% of gender differences in stress levels and 31% in depression levels. The economic field mediated both stress and depression levels by 13% and 25%, respectively.

## 4. Discussion

The aim of this study was to examine the prevalence of depressive symptoms and the level of perceived stress in Slovak students during ongoing e-learning. Our first hypothesis assumed that Slovak students presented symptoms of stress and depression. Analysis of the results confirmed the hypothesis as the mean score for stress (PSS-10) of the study group was 20.85 (SD 5.63). Forty-seven percent of participants are prone to depressive symptoms and nine percent have severe depression. Additionally, the mean score for depression (BDI) was 14.35 (SD 10.22). This suggests that participants may be prone to moderate mood disorders. These results are significantly higher compared to a study conducted on Slovakian students before the outbreak of the pandemic, where depressive symptoms appeared in 35.5% of participants [22]. The results of a study performed during a pandemic among college students in the United States showed that increased levels of fear and stress were associated with academic factors, primarily with concerns about grades and the future, difficulties in adapting to distance learning, decreased opportunities for additional work, and financial difficulties [29]. These results are in line with our findings. As for major problems, the participants indicated the reduction in financial resources (53%), the limited ability to work (56%), and worrying about practical skills (62%). 

The second hypothesis assumed that mental health disorders affect women more than men. Analysis of the result confirmed the hypothesis. Women were much more affected by e-learning and social isolation. The mean scores for stress and depression were higher in women. Moreover, 47% of women presented a very high level of stress (compared to 30% of men) and 11% showed severe depressive symptoms (compared to 7% of men). The results of the author’s survey suggest that women were much more affected by the reduction of financial resources, the limited ability to work, and increased conflicts within the family. Our findings are in line with the results of Marcén-Román et al. that indicate that 14.6% of women experienced increased stress. Moreover, women are almost 10 times more prone to have elevated stress levels compared to men [34]. 

The third hypothesis assumed a negative impact of the pandemic and e-learning implementation on four areas of life: social life, educational skills, economic field, and nutritional and drug habits. Analysis of the results partially confirms this hypothesis; a significant effect was noted in the social and economic fields. The mediation analysis showed that women were characterized by a higher severity of depressive symptoms and stress levels within these areas. 

The explanation for such dependencies is sought in the differences between women and men. Physiological differences (e.g., hormone levels) can be reflected in behavior and emotions. Women tend to be more emotional, compassionate, and empathetic. Therefore, in difficult moments women more often struggle with negative emotions and feelings such as anxiety and depression. On the other hand, men are more assertive individualists [35,36,37]. Thus, men try to solve their problems through the abuse of different substances (e.g., tobacco, alcohol, drugs) [36,37]. The presented relationships and results indicate that it is necessary to improve the mental health of students. However, it is important to adjust the methods of intervention for women and men separately, because each of them has different problems. Women’s mental health should be supported early for the reason of motherhood. Postpartum depression affects approximately 10–15% of women and impairs mother-infant interactions that in turn are important for child development [38].

The presented findings lead to reflection on the importance of managing mental health, especially in young people who are constantly preparing themselves to fulfill new social roles. Scott et al. showed that mental distress in early adulthood is associated with long-term complications in later adulthood, including persistent health problems and emotional and physical relationship disorders [39]. Thus, a global lockdown may cause the development of numerous mental health disorders and, as a result, lead to many problems later in life. There are many strategies for coping with problems associated with isolation. Some young people more often use stimulants (e.g., alcohol, cigarettes), while others have taken additional physical activity or further develop their hobbies [40,41]. Unfortunately, some young people confirm that they cannot cope with the stress related to the current situation. Therefore, some of them experience suicidal thoughts as well as those of self-harm [21,42]. The result obtained by Marcén-Román et al. shows that almost a year after the start of the pandemic, 13.1% of the university students perceived higher stress levels [34]. Interestingly, this is less than at the start of the pandemic, when 28.14% of the surveyed students were prone to stress symptoms [43]. This is most likely related to increased awareness about the whole situation related to COVID-19. However, scientists agree that the issue of mental well-being among students should be immediately taken into account [21,44]. Even before the pandemic period it was pointed out that universities should take measures to support students, especially in the realm of mental health [44]. 

Our study is the first to comprehensively assess mental health status in a population by gender. The areas of life that have the greatest influence on the symptoms of depression and stress have also been identified. The results may be a universal conclusion and provide grounds for political and social actions in other countries as well. Introducing appropriate support programs for students could have the potential to improve their psychological condition. The results of our study suggest that support activities should focus primarily on women. Properly selected and performed emotional training could improve the mental well-being of women. This is of great importance in the process of preparing them to fulfill new social roles (e.g., motherhood). It is believed that negative childhood experiences increase pregnancy-related anxiety and negatively affect the acceptance of the parenthood role [45]. Thus, it is possible that negative experiences and emotions in early adulthood may also contribute to concerns about motherhood. Appropriate psychological support would also be important for men, who often use various stimulants in stressful situations. Coping with negative emotions in the present pandemic era seems to be a key issue that will have a significant impact on the future of people. Therefore, it seems that the activities of the university can become crucial in socially shaping young adults.

## 5. Conclusions

To conclude, the worldwide lockdown has affected many aspects of human health. The findings of our study confirm that mental well-being is one of the most serious problems related to the COVID-19 pandemic among students. Depression and stress became a worldwide pandemic issue. As these factors could significantly affect the overall health in subsequent years, it is important to properly manage the mental health of people, regardless of age. Moreover, more efforts should be made to create programmes for students during e-learning periods to promote healthy lifestyles. 

The attention of the university management should be directed at the changes in the functioning of the mental health supports. Students should receive support in managing the new situation in education, and this should focus primarily on stress reduction and negative emotions control, such as by providing access to relaxation programs and educational materials on mental health prevention. Universities should increase the working hours of professionals such as psychologists who provide direct assistance to students in difficult situations. Special attention should be given to women and their mental health needs.

The restoration of mental health may prevent the development of further diseases and facilitate the return to normalcy after this difficult COVID-19 period. Caring for the mental health of young people is an investment in the future in the functioning of the country.

## Figures and Tables

**Table 1:** Authors’ survey areas and questions.

Area	Question
Social life	E-learning isolated me from my friends.
	The pandemic has caused increased conflicts within the family.
	The pandemic significantly limited my social contacts.
Education skills	E-learning has had a negative impact on my level of knowledge.
	E-learning has had a negative impact on my level of practical skills.
	E-learning reduced my motivation to learn.
	I think that I will get worse credits at exams due to distance learning.
Economic field	The pandemic prevented me from taking up additional work.
	The pandemic has reduced my financial resources.
Nutrition habits and drugs	I use more stimulants (cigarettes, alcohol, drugs) during the pandemic.
	The pandemic has had a negative effect on my diet.

**Table 2:** Participant’s characteristics.

		Total (*n* = 3051)
% females		58.11
Age (years), mean (SD)		22.37 (4.22)
Body height (cm), mean (SD)		173.14 (9.55)
Body mass (kg), mean (SD)		69.61 (15.05)
BMI, mean (SD)		23.08 (3.86)
Chronic diseases, n (%)		435 (14.26)
Taking medicines, n (%)		667 (21.86)
Will to seek the help of a psychologist, n (%)		1881 (61.7)
Prefering e-learning, n (%)		451 (14.8)
Type of studies		
	Bachelor	2152 (70.53)
	Master	871 (28.55)
	PhD	28 (0.92)
Year of studies:		
	First year undergraduate	1189 (38.97)
	Second-year undergraduate	847 (27.76)
	Third-year undergraduate	536 (17.57)
	Fourth-year undergraduate	249 (8.16)
	Fifth-year undergraduate	230 (7.54)
Method of education		
	E-learning	2896 (94.92)
	Mixed form (hybrid)	105 (3.44)
	In-class study	50 (1.64)

BMI: Body Mass Index; SD: Standard Deviation.

**Table 3:** Means (SDs) and statistical differences in the mental state between women and men.

	Total (*n* = 3051)	Women (*n* = 1773)	Men (*n* = 1278)	*p* Value *
PSS-10, mean (SD)	20.85 (5.63)	21.97 (5.39)	19.30 (5.59)	<0.001
BDI, mean (SD)	14.35 (10.22)	15.43 (10.20)	12.85 (10.05)	<0.001
Authors survey	48.97 (10.68)	49.64 (10.22)	48.03 (11.23)	<0.001

* according to unpaired *t* test.

**Table 4:** Authors survey results with the specification of individual areas.

Area	Gender	Mean (SD)	*p* Value *	(1)	(2)	(3)	(4)	(5)
Sense of isolation	Women	3.72 (1.30)	0.004	9%	15%	4%	39%	33%
	Men	3.58 (1.33)		9%	19%	7%	35%	30%
Worsening of knowledge	Women	3.12 (1.29)	0.054	11%	32%	7%	36%	15%
	Men	3.22 (1.34)		13%	25%	10%	34%	19%
Worsening of practical skills	Women	3.75 (1.26)	0.002	7%	16%	9%	35%	34%
	Men	3.60 (1.37)		11%	16%	8%	32%	33%
Recuced learning motivation	Women	3.32 (1.44)	0.23	14%	23%	7%	29%	28%
	Men	3.27 (1.47)		16%	22%	8%	26%	27%
Deteriorated quality of education	Women	3.62 (1.23)	<0.001	6%	18%	10%	39%	27%
	Men	3.41 (1.32)		10%	19%	14%	33%	24%
Limitation of work	Women	3.57 (1.46)	<0.001	14%	13%	11%	24%	38%
	Men	3.21 (1.49)		20%	15%	15%	22%	27%
Reduced financial resources	Women	3.46 (1.47)	<0.001	15%	17%	11%	23%	34%
	Men	3.12 (1.49)		20%	20%	14%	21%	26%
Increased supply of drugs	Women	1.78 (1.23)	0.49	63%	16%	5%	10%	5%
	Men	1.81 (1.26)		62%	16%	5%	10%	6%
Negative impact on diet	Women	2.66 (1.45)	0.49	28%	30%	5%	23%	14%
	Men	2.63 (1.45)		31%	25%	9%	22%	14%
Increased conflicts within family	Women	2.90 (1.43)	<0.001	22%	25%	9%	27%	16%
	Men	2.60 (1.37)		28%	27%	11%	23%	10%
Limited social contact	Women	4.19 (1.10)	0.007	4%	10%	2%	33%	51%
	Men	4.07 (1.21)		6%	10%	4%	31%	49%

* according to unpaired *t* test; (1) I strongly disagree; (2) I rather disagree; (3) I have no opinion; (4) I rather agree; (5) I strongly agree.

**Table 5:** Mediation analysis.

	Total Effect		Direct Effect		Indirect Effect		Percentage Mediation
	Effect Size (95% CI)	*p* Value	Effect Size (95% CI)	*p* Value	Effect Size (95% CI)	*p* Value	
PSS-10							
Social life	−2.67 (−3.06, −2.27)	<0.001	−2.18 (−2.54, 1.83)	<0.001	−0.49 (−0.68, −0.30)	<0.001	18.35
Economic field			−2.31 (−2.69, −1.93)	<0.001	−0.36 (−0.48, −0.25)	<0.001	13.48
BDI							
Social life	−2.58 (−3.32, −1.86)	<0.001	−1.79 (−1.12, −0.17)	<0.001	−0.79 (−1.10, −0.48)	<0.001	30.62
Economic field			−1.94 (−2.65, −1.22)	<0.001	−0.64 (−0.84, −0.45)	<0.001	24.80

BDI: Beck depression inventory; CI: Confidence interval; PSS-10: Perceived Stress Scale.

## Data Availability

Data is available upon request from the corresponding author.
